# 

**Published:** 1886-12-25

**Authors:** 


### The Hospital.

An Institution, Family, and Parochial Journal of Hospitals, Asylums, and all Agencies for the Care of the Sick, Criticism and News.

SATURDAY, DECEMBER 25TH, 1886.

### The In- and Out-patients' Hospital Diary.

This Diary is so arranged as to make some portion of it appear every week, and the whole in each monthly part. It has been very difficult to compile, and corrections will at all times be gratefully received. It has been suggested that similar information about the larger Provincial and Scotch Hospitals would be useful to many readers. We should be glad to hear if this is felt to be the case.

### DISEASES AND IRREGULARITIES OF THE TEETH.—*continued.*

| Hospitals and Terms of Admission. | Physicians, Surgeons, and Medical Officers, with Days and Hours of Attendance. |
|---|---|
| New Hospital for Women, 222, Marylebone rd., W. | *Surgeon*, Mr. H. Apperley |
| North-West London Hosp., 18, Kentish Town Rd., N.W. | *Surgeon*, Mr. W. A. Maggs, F. 9 |
| Paddington Green Children's Hospital, Paddington | *Surgeon*, Mr. C. Vincent Coterell, W. 9 |
| Royal Free Hospital, Gray's Inn Road, W.C. | *Surgeon*, Mr. H. Harris, M. Th. |
| Royal Hospital for Children and Women, Waterloo Bridge Road, S.E. | *Surgeon*, Mr. Whitehouse, F. 1.30 |
| St. Bartholomew's Hospital, Smithfield, E.C. | *Surgeons*, Messrs. Ewbank, Tu.; Paterson, F.; Ackery, F.; Mackrett, Tu. Hour, 9 |
| St. George's Hospital, Hyde Park Corner, S.W. | *Surgeon*, Mr. Winterbottom, Tu. S. 9 |
| St. Mary's Hospital, Cambridge Place, Paddington | *Susgeon*, Mr. H. Hayward, W. S. 9.30 |
| St. Thomas's Hospital, Westminster Bridge Road, S.E. | *Surgeon*, Mr. Truman, Tu. F. 10 |
| University College Hospital, Gower Street, W.C. | *Surgeon*, Mr. S. J. Hutchinson, W. 9.30 |
| Victoria Hospital for Children, Chelsea, S.W. | *Surgeon*, Mr. F. Fox, S. 9.0 |
| West London Hospital, Hammersmith Road, W. | *Surgeon*, Mr. H. L. Albert, Tu. F. 9.30 |
| Westminster Hospital, Broad Sanctuary, S.W. | *Surgeons*, Messrs. J. Walker and M. A. Smale, W. S. 9.15 |

### THROAT DISEASES.

| | |
|---|---|
| Central London Throat and Ear Hospital, Gray's Inn Road, W.C. | *Surgeons*. *See* "Ear Cases." M. W. Th. S. 2 30; Tu. F. 5 |
| Great Northern Central Hospital, Caledonian Rd, N. | *Surgeon*, Mr. Spencer Watson, W. 2 |
| Guy's Hospital, St. Thomas Street, S.E. | *Surgeon*, Mr. Symonds, Th. 1 |
| Hospital for Diseases of the Throat and Chest, 32, Golden Sq. Hon. Sec., Mr. G. C. Witherby. Poor free; others by small payment | *Physicians*, Drs. Wolfenden, Tu. F. 2.30; Macdonald, Tu. F. 6 30; Bond, W. S. 2.30<br>*Surgeon*, Mr. T. Mark Hovell, M. Th. 2.30 |
| King's College Hospital, Portugal Street, W.C. | *Surgeon*, Mr. Cartwright, Tu. F. 10 |
| Middlesex Hospital, Berners Street, W. | *Surgeon*, Mr. Hensman, Tu. 9 |
| North West London Hospital, 18, Kentish Tn. Rd., N.W | *Surgeon*, Mr. Collier, M. 1.30 |
| St. Bartholomew's Hospital, Smithfield, E.C. | *Surgeon*, Mr. Butlin, F. 2.30 |
| St. George's Hospital, Hyde Park Corner, S.W. | *Surgeon*, Dr. Whipham, Th. 1.30 |
| St. Mary's Hospital, Cambridge Place, Paddington | *Surgeon*, Mr. A. T. Norton, M. Th. 9.30 |
| St. Thomas's Hospital Westminster Bridge Road, S.E. | *Physician*, Dr. Semon, Tu. F. 1.30 |
| University College Hospital, Gower Street, W.C. | *Physician*, Dr. Poore, Th. 1.30 |
| Westminster Hospital, Broad Sanctuary, S.W. | W. 9. *See* General Hospitals. |

### DISEASES OF WOMEN, (OBSTETRIC CASES, ETC.)

| | |
|---|---|
| Charing Cross Hospital, West Strand, W.C. | *Physicians*, Drs. J. W. Black, Amand Routh, Tu. F. 1.30 |
| Chelsea Hospital for Women, Fulham Road, S.W. Sec., Mr. Easterbrook. Payment from 10*s*. 6*d*. a week. Poor by Governor's letter. Out-patients, if without subscriber's letter, pay 6*d*. each attendance | *In-Physicians*, Drs. J. H. Aveling, M. Th.; A. W. Edis, Tu. F.; F. Barnes, W. S. Hour, 2.30<br>*Out-Physicians* Drs. Dickinson and Mackeron, M.; W. Travers and G. Harper, Tu.; F. Jones and J. I. Parsons, W.; H. T. Rutherford, and A. Miller, F. Hour, 2 |

### DISEASES OF WOMEN, (OBSTETRIC CASES, ETC.)—*Continued.*

| Hospitals and Terms of Admission. | Physicians, Surgeons, and Medical Officers, with Days and Hours of Attendance. |
|---|---|
| East London Hospital for Children and Dispensary for Women, Shadwell, E. A Subscriber's letter is required, except in urgent cases | *Out-Physicians*, M. Tu W. Th. F. 1 and 3; Tu. F. 9. *See* "Children's Diseases." |
| German Hospital, Dalston Lane, Dalston, E. | *Physician*, Dr. Rasch, M. Th. 9 |
| Great Northern Central Hospital, Caledonian Rd., N. | *Physician*, Dr. F. Barnes, W. 2 |
| Grosvenor Hospital for Women and Children, 3 and 4, Vincent Square, S.W. | *Out-Physicians and Surgeons*, Tu. W. Th. S. 2. *See* "Children's Diseases." |
| Guy's Hospital, St. Thomas Street, S.E. | *Physicians*, Drs. Galabin, Horrocks, M. Tu. F. 1.30 |
| Hospital of St. John and St. Elizabeth, 47, Gt. Ormond Street, W.C. For females suffering from long standing disease. | *Physicians*, Drs. Constable and Tegart (no out-patients) |
| Hospital for Women, 29 and 30, Soho Sq., W. Sec., Mr. David Cannon. In-patients admitted free by Governor's letter; also by payment. Out-patients free | *In-Physicians*, Drs. Carter, W. S. 2; R. T. Smith, Tu. F. 2; E. Holland, Tu. F. 9.30<br>*In-Surgeon*, Mr. H. A. Reeves, M. 4, Th. 2;<br>*Out-Physicians*, Drs. R. T. Smith, F. 10; E. Holland, W. 10; Mansell-Moullin, M. Th. 11; Bedford Fenwick, Tu. 10; J. Oliver, S. 10<br>*Out-Surgeon*, Mr. S. Osborn, Th. 2 |
| King's College Hospital, Portugal Street, W.C. | *Physician*, Dr. Playfair, Tu. Th. S. 2 |
| London Hospital, Whitechapel Road, E. | *In-Physician*, Dr. Herman, Tu. F. 1.30<br>*Out-Physician*, Dr. Lewers, W. S. 1.30 |
| Metropolitan Free Hospital, Gray's Inn Road, W.C. | *Physician*, Dr. A. Venn, W. 2 |
| Middlesex Hospital, Berners Street, W. | *In-Physician*, Dr. Edis, Tu. F. 1.30<br>*Out-Physician*, Dr. Duncan, W. S. 1.30 |
| New Hospital for Women, 222, Marylebone Road, W. Hon. Sec., Miss C. Vincent. By subscriber's letter. Outpatients pay 6*d*. entrance fee, and 2*d*. each visit. | *In-Physicians*, Drs. Anderson, Atkins, Marshall, de la Cherois<br>*Out-Physicians*, Drs. Marshall, De la Cherois, Hitchcock. Daily, 1 to 3, and S 9 to 10. (All the physicians are women) |
| North-West London Hospital, 18, Kentish Tn. Rd., N.W | W. 1.30. *See* General Hospitals |
| Royal Free Hospital, Gray's Inn Road, W.C. | *In- and Out-Physician*, Dr. Hayes, Tu. S. 9 |
| Royal Hospital for Children and Women, Waterloo Bridge Road, S.E. Sec., Mr. R. G. Kestin. | *Out-Physicians*. Daily at 2<br>*Out-Surgeon*, W. 2<br>*See* "Children's Diseases." |
| St. Bartholomew's Hospital, Smithfield, E.C. | *In-Physician*, Dr. M. Duncan, Tu. Th. 2<br>*Out-Physician*, Dr. Godson. W. S. 9 |
| St. George's Hospital, Hyde Park Corner, S.W. | *Physician*, Dr. Champneys, Th. 1.30 |
| St. Mary's Hospital, Cambridge Place, Paddington. W. | *Physicians*, Drs. Meadows, Tu. F. 9.30; H. Jones, M. Th. 1.30 |
| St. Thomas's Hospital, Westminster Bridge Road, S.E. | *Physician*, Dr. Jervis, Tu. F. 2 |
| Samaritan Free Hospital for Women and Children, Lower Seymour Street, W. Branch, 1, Dorset Street, Manchester Square, W. Sec., Mr. Geo. Scudamore. Governor's letter or card is required if the disease of the applicant is *not* peculiar to her sex. Out-patients department (free) is at Edward's Mews, Duke Street, W. | *In-Physicians*, Drs. P. Boulton, M. Prickett, Amand Routh. Daily, 2<br>*In-Surgeons*, Messrs. G. G. Bantock, J. H. Thornton, W. A. Meredith. Daily. 9.30<br>*Out-Physicians*, Drs M. Prickett, and Amand Routh, W. S. 1.30<br>*Out-Surgeons*, Messrs. W. A. Meredith, and J. D. Malcolm, M. Th.; A. H. G. Doran, and W. S. A. Griffith, Tu. F. Hour 1.30 |
| University College Hospital, Gower Street, W.C. | *Physicians*, Drs. G. Hewitt, M. Th. 1.30; J. Williams, Tu. F. 2 |
| West London Hospital, Hammersmith Road, W. | *Physicians*, Drs. A Venn, Moullin, W. S. 2 |
| Westminster Hospital, Broad Sanctuary, S.W. | *Physicians*, Drs. Potter, Tu. F. 2; Grigg, Tu. F. 9 |

### THE CHILDREN'S CORNER.

### OUR PASTIMES.

**Children's Quarterly Prize Competition.**

Began .. .. .. .. .. 2nd Oct. 1886
Ends .. .. .. .. .. 25th Dec., 1886

PRIZES.

1st .. .. .. .. .. Thirty Shillings

2nd .. .. .. .. .. Twenty „

3rd .. .. .. .. .. Ten „

4th .. .. .. .. .. Five „

will be awarded to the four competitors who shall have sent in, during the thirteen weeks, the largest number of correct answers to our pastimes.

ANOTHER week and the victory will be won! So close are some of my little friends in the race, that even now the scale may turn. Therefore be sure you make a supreme effort in the Thirteenth Competition, and send me as many correct answers as you possibly can.

**Thirteenth Competition.**

No. 68. RIDDLE-ME-REE.

My *first* is in pin, but not in sin,
My *second's* in tray, but not in way,
My *third* is in tie, but not in why,
My *fourth* is in zone, but not in tone,
My *fifth* is in frowned, but not in sound.

No. 69. BURIED NAMES. The following letters arranged aright, will give you the name of a popular English minister.

b r y a s s u l i

No. 70. AN ARITHMETICAL PROBLEM. A fish had a head three feet long; its tail was as long as its head and half of its body; its body was as long as its head and its tail put together. What was the length of the whole fish?

No. 71. PUZZLE SENTENCE.

u
come
dangers by 40 tude.

No. 72. A QUESTION OF SIMILARITY. Who amongst my little friends can tell me why heiresses whose wealth is protected by Chancery, resemble the rooms of a hospital where the in-patients lie?

No. 73. A QUESTION IN SUBTRACTION. Two men went out shooting, and saw ten birds on a tree. One man killed two of them, and the other, three. Now, tell me how many remained on the tree alive?

No. 74. PYRAMID PUZZLE.

```
    * *
   * * *
  * * * * *
 * * * * * * *
* * * * * * * * *
```

Two of the cardinal points; a river of Sweden; an agency which telegraphs news from abroad; a word meaning to encircle; people who place things on view. The pyramid read down one side gives a person we are glad to see in time of sickness; read down the other side the word means divisions of a hospital.

**Results of Eleventh Competition.**

No. 55. Master Jimmy has alone accomplished this little problem. The first sketch shows the two cuts to be made; the second gives the three pieces set in the form of a square:

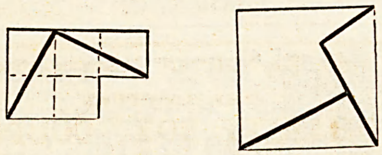

No. 56. Some ingenious little guesses have been made here, but only Diosota and True Blue have solved the question right. First of all let us take

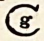

This is a "g" (pronounced *zhay*) in (=*dans*) the letter "c" (pronounced *say*), so we get *J'ai dansé*. Now this is placed *under* some *o's ranged along*, or, in French, *sous les o's rangés*. Hence we get the little sentence to read, "J'ai dansé sous les orangers" (=I have danced under the orange trees). Tina suggests as a translation, "J'ai mangé avec les héros," which is by no means bad; while Retsibsi writes, "J'ai dansé sous les étoiles." I was surprised not to see Alsie attempt it.

No. 57. Here was an easy one which everybody guessed. The most difficult train to catch is, of course, the 12.50, because 'tis *ten to one*, if you catch it.

No. 58. This was nearly guessed by everyone, but, curiously enough, no one succeeded in getting it quite right.

H
nOt
paSte
samPles
ASSOCIATION
LaTin
fAr
L

No. 59. DOUBLE ACROSTIC. The *lights* here are

D o M
O k A
C a T
T a R
O h O
R u N

Two or three competitors give "Onega" as the second light, and, as there is a river of this name in Russia, I of course accept it, but the Oka (a tributary of the Volga) was the one intended.

No. 60. The Children of Israel could not have starved in the desert because of the sandwiches there (sand which is there), for Noah sent *Ham* into the desert and his descendants bred (*bread*) and mustered (*mustard*)!

Four right—Little Mary.
Three right—Tina, Alsie, Jimmy, Florrie, Retsibsi, and T. K.
Two right—Mikado, True Blue, Filomel, Diosota, Bismarck.

Answers, having the actual name and address (to which a pseudonym may be added for publication), must have "The Children's" Coupon attached (see advertisement pages), and be addressed to the "PUZZLE EDITOR," THE HOSPITAL, Norfolk House, Norfolk Street, Strand, W.C., not later than Thursday, 30th December. Results will appear in our issue of the 8th January, 1887.

### "THE HOSPITAL" CHARITY BOX.

OUR "Charity Box" is intended to afford a little practical help to the hospitals. Each week we propose a word for "anatomical dissection," and the first 150 competitors who extract the largest number of words from it receive the following prizes:—

1st .. .. .. One Guinea.
2nd .. .. .. Half a Guinea.
3rd .. .. .. Five Shillings.

The above Prizes will be repeated for every additional 150 competitors who may enter each week

THIRTEENTH COMPETITION.

Compose the largest number of words you can from "DISPENSER."

*Observe:* Proper names, abbreviations, foreign, Latin, and obsolete words are barred.

Repetitions of similarly spelt words and of letters (unless they repeat in the word) are disallowed.

Plurals allowed, but not the possessive in 's or s'.

Prefixes and affixes do not count independently of words.

Write only on one side of the paper, and total your words.

Append your real name and address, adding whether Mr., Mrs., or Miss, etc.

All replies, addressed "Charity Box," THE HOSPITAL, Norfolk House, Norfolk Street, W.C., must arrive by Friday, Dec. 31st. Results will appear Saturday, Jan 8th, 1887.

*Every Competitor is required to cut out and enclose with his list the "Charity Box" Coupon* (see advertisement pages), *and a shilling entrance fee* (postal orders only, *crossed Lloyd's, etc., Banking Company, Limited,* THE HOSPITAL *Account.*)

The proceeds resulting from each "Charity Box" competition will be funded until the end of December 1886. The total sum available for distribution, and the names of the hospitals selected for donations, will appear on Saturday, January 8, 1887. The distribution of the proceeds will be at the Editor's sole discretion.

RESULTS OF ELEVENTH COMPETITION

The "Physician" Competition has resulted as follows:—

| | Accepted Score. |
|---|---|
| Miss Powell, 21, Grosvenor Place, Margate .. | 146—1st Prize. |
| Miss Gore, Fellenburg House, Jersey .. .. | 141—2nd Prize. |
| Mr. W. Jameson, 111, Bushey Hill Road, Camberwell .. .. .. .. .. .. .. | 133—3rd Prize. |

The prizes have been forwarded to the winners.

**Special Notice.**

In the interest of non-successful competitors, we have decided not to award the First Prize more than once to a winner in any one quarter. In the event of a competitor again making the highest accepted score, he will be placed in the second place, and, if a third time highest, in the third place.

We must particularly ask correspondents not to swell their lists by the inclusion of purely Latin words, and abbreviations.

**Notice to Correspondents.**

Erica.—"Oc" signifies an arrow. It occurs in Annandale's edition of *Ogilvie.*

